# Staging laparoscopy with ultrasound and near-infrared fluorescence imaging to detect occult metastases of pancreatic and periampullary cancer

**DOI:** 10.1371/journal.pone.0205960

**Published:** 2018-11-01

**Authors:** H. J. M. Handgraaf, B. G. Sibinga Mulder, S. Shahbazi Feshtali, L. S. F. Boogerd, M. J. M. van der Valk, A. Fariña Sarasqueta, R. J. Swijnenburg, B. A. Bonsing, A. L. Vahrmeijer, J. S. D. Mieog

**Affiliations:** 1 Department of Surgery, Leiden University Medical Center, Leiden, The Netherlands; 2 Department of Radiology, Leiden University Medical Center, Leiden, The Netherlands; 3 Department of Pathology, Leiden University Medical Center, Leiden, The Netherlands; University of Toronto, CANADA

## Abstract

**Introduction:**

Up to 38% of pancreatic and periampullary cancer patients undergoing curative intended surgery turn out to have incurable disease. Therefore, staging laparoscopy (SL) prior to laparotomy is advised to spare patients the morbidity, inconvenience and expense of futile major surgery. The aim of this study was to assess the added value of SL with laparoscopic ultrasonography (LUS) and laparoscopic near-infrared fluorescence imaging (LFI).

**Methods:**

All patients undergoing curative intended surgery of pancreatic or periampullary cancer were included prospectively in this single arm study. Patients received an intravenous infusion of 10 mg indocyanine green (ICG) one or two days prior to surgery to allow LFI. Suspect lesions were analyzed via biopsy or resection. Follow-up visits after surgery occurred every three months.

**Results:**

A total of 25 patients were included. Suspect lesions were identified in 7 patients: liver metastases (n = 2; identified by inspection, LUS, and LFI), peritoneal metastases (n = 1; identified by inspection only), and benign lesions (n = 4; identified by inspection or LUS). Quality of LFI was good in 67% (10/15) of patients dosed one day and 89% (8/9) dosed two days prior to surgery. A futile laparotomy was averted in 3 patients (12%). Following SL the primary tumor was resected in 20 patients. Two patients (10%) developed metastases within 3 months after resection.

**Conclusions:**

Despite current preoperative imaging modalities metastases are still identified during surgery. This study shows limited added value of LUS during SL in patients with pancreatic or periampullary cancer. LFI was of added value due to its high negative predictive value in case of suspect hepatic lesions identified by inspection.

## Introduction

Pancreatic and periampullary cancers are dreadful diseases with a poor prognosis. At time of diagnosis only 10% to 20% of pancreatic cancer patients is eligible for curative intended surgery, but during explorative laparotomy up to 38% of those patients turn out to have distant metastases or an unresectable primary tumor [1]. Yet, even after resection with curative intend, 5-year survival rates are still disappointingly low between 6.8% and 32% [2]. Up to 70% of patients with resectable pancreatic cancer suffer from distant metastases, of which the majority occurs within 6 months after surgery [3]. These metastases may have been present during surgery without being detected. Preoperative imaging modalities, including computed tomography (CT) and magnetic resonance imaging (MRI), have low sensitivity for subcentimeter peritoneal and liver metastases [4]. Especially superficial metastases are difficult to detect. In a disease with such a dismal prognosis, it is important to spare patients with incurable disease the morbidity, inconvenience and expense of futile major surgery.

The Society of American Gastrointestinal and Endoscopic Surgeons (SAGES) advocates that staging laparoscopy (SL) should be considered in selected patients [5]. Compared to explorative laparotomy, SL results in less postoperative pain, a shorter hospital stay and chemo- and/or radiotherapy can be administered more often and sooner to patients [6]. The chance of an unnecessary laparotomy in patients who appear eligible for curative resection based on preoperative imaging decreases from 40% to 17% by performing SL [7]. Moreover, a laparotomy is not required nowadays in case of palliative treatment; metal stents make biliary anastomosis unnecessary and other bypass surgery can be done laparoscopically.

The yield of SL may be amplified by adding laparoscopic ultrasonography (LUS) and laparoscopic near-infrared fluorescence imaging (LFI). LUS enables identification of metastases located deep in the liver and, additionally, vascular involvement of the primary tumor can be assessed [8]. Open-space fluorescence imaging or LFI using indocyanine green (ICG) is a safe and easy method to identify microscopic (sub)capsular liver metastases not yet visible by any other means [9]. Yokoyama et al. previously demonstrated that open space fluorescence imaging is able to identify additional micrometastases in the liver in 16% of the patients with pancreatic cancer [10].

Although selection of patients for SL is being advised, there is no scientific support, nor a consensus on which selection criteria should be used [11, 12]. The current study combines SL with LUS and LFI and aims to determine the added value of these three modalities in all patients with pancreatic and periampullary cancer before undergoing surgery with curative intent.

## Methods

### Patients

This single arm, open label, single center clinical study protocol (S1 File) was approved on November 29 2015 by the medical ethics review board (*‘Commissie Medische Ethiek’*) of the Leiden University Medical Center (LUMC) and conducted in concordance with the Helsinki Declaration of 1975 (as amended in Tokyo, Venice, Hong Kong, Somerset West, Edinburgh, Washington, and Seoul), ICH-GCP guidelines, and the laws and regulations of the Netherlands. The study protocol has been registered at the Netherlands National Trial Register (registry number NTR6639) after enrollment of participants started, due to an administrative error. This non-randomised study adheres to the Transparent Reporting of Evaluations with Non-Randomised designs (TREND) guidelines (S1 Fig). The authors confirm that all ongoing and related trials for this drug/intervention are registered. All subjects provided written informed consent prior to the start of any study-related procedure. All patients of 18 years or older undergoing resection of suspected pancreatic or periampullary cancer at the LUMC were eligible for inclusion. Exclusion criteria were either participation in other clinical trials or contraindications for ICG administration: eGFR <55; hyperthyroidism; and allergy to iodine, shellfish or ICG. Included patients received standard-of-care, including pancreas-specific CT utilizing a thin-section, multi-phase technique with pancreatic phase and portal venous phase images. All patients received water as oral contrast. Additional imaging, for example contrast-enhanced MR with 3D-MRCP, (endoscopic) ultrasound or FDG-PET, was performed if deemed necessary by the multidisciplinary team. Patients who did not provide informed consent or those who were included in other trials still received SL, but without LUS and LFI.

### Staging procedure

Patients received 4 mL 2.5 mg/mL (10 mg in total) ICG one day prior to surgery to allow intraoperative LFU of the liver surface. This dose and dosing time was based on previous experiences [13]. Adverse events where collected according to the local protocol and graded using the Clavien-Dindo classification. Before laparotomy, SL was performed via two ports of 10 mm and one port of 5 mm: a subumbilical port and two ports along the planned laparotomy line. Laparoscopic inspection of the abdomen was performed, including the parietal and visceral peritoneum, the pelvis, the liver, the porta hepatis, the gastrohepatic omentum, the duodenum, the transverse mesocolon and celiac region. Second, LUS (with or without Doppler; Toshiba Aplio 300, with a laparoscopic probe) of the liver was performed by a trained surgeon. LFI of the liver surface was performed lastly using a high-definition fluorescence imaging system (Karl Storz GmbH & Co. KG, Tuttlingen, Germany). Lesions with a fluorescent rim were considered suspect. Quality of fluorescence imaging was divided into three categories: good, meaning that healthy liver was dark and bile ducts and/or intestines were fluorescent; medium, meaning that healthy liver showed some remaining fluorescence; or bad, meaning that the liver was either totally fluorescent or totally dark and no fluorescence was seen in bile ducts nor intestines. The categories medium and bad were considered insufficient for adequate fluorescence imaging. We hypothesized that cholestasis would decrease the quality of fluorescence imaging at one day after ICG administration. If so, dosing time would be extended to two days before surgery.

Any lesions suspect for metastases based on inspection, LUS or LFI were sampled and analyzed, either by biopsy or by resection. In case of multiple lesions with similar appearance on inspection, LUS and LFI, only one biopsy was taken. Histopathological examination was considered the gold standard. The surgical procedure continued via laparotomy if the tumor appeared to be resectable and no metastases were identified.

### Follow-up

Follow-up occurred according to the local standard protocol, including a visit every three months to the surgical outpatient clinic of surgery in conjunction with the department of oncology. A CT-scan was performed only if locoregional or metastatic disease was suspected.

### Outcomes

Main outcome of this study was the percentage of averted futile laparotomies. Secondary outcome was the accuracy of the diagnostic modalities. Findings of SL, LUS, LFI were compared with histopathological examination of intraoperative biopsies, findings after laparotomy and finally, with follow-up results until at least the first visit to the outpatient clinic (i.e. approximately 3 months after surgery).

### Statistics

Using A’Hern’s single-stage phase II trial design and alpha = 0.05 and power = 80%, 25 patients were needed to distinguish between an averted laparotomy rate of 30% (worth exploring in a Phase III trial) and 10% or less (unacceptable outcome) [14]. This required at least six averted laparotomies to reach the positive endpoint. Statistics were calculated using SPSS (version 23.0, IBM Statistics, US).

## Results

### Patient characteristics

**Fig 1:**
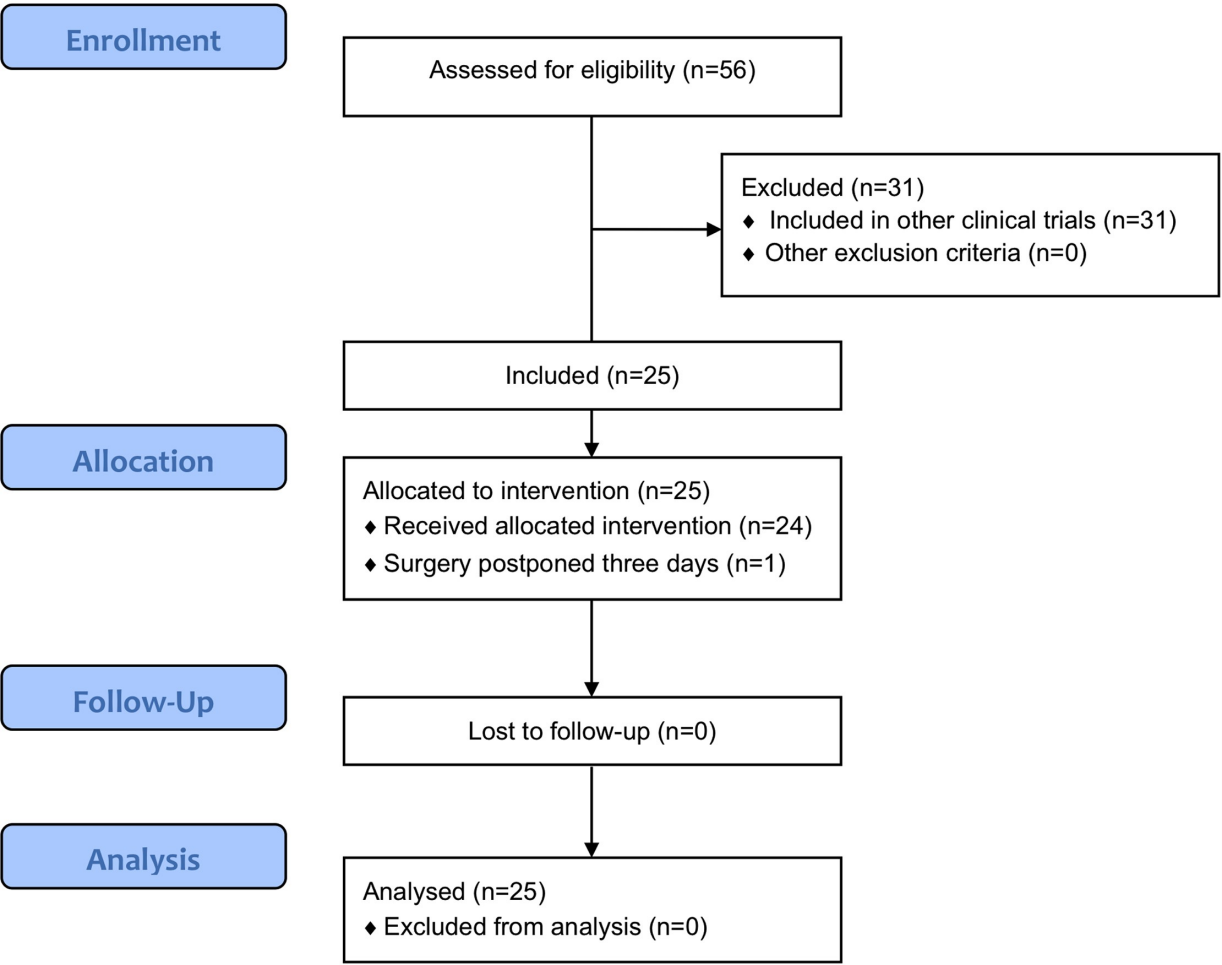
CONSORT flow diagram.

Twenty-five patients were included between *January* 2016 and April 2017 (Fig 1 and S2 File). Patient and tumor characteristics are summarized in Table 1. Three patients received neoadjuvant therapy. One patient was treated with gemcitabine and radiotherapy, two patients with FOLFIRINOX. In one patient a liver metastasis was already suspected based on preoperative imaging, but no histopathological diagnosis could be obtained. During SL a liver metastasis was confirmed by histopathological examination. None of the patients experienced adverse events after ICG administration. Post-operative complications did occur, but all were assessed as related to resection of the primary tumor rather than to the SL.

**Table 1:** Patient and tumor characteristics.

Gender, % (n)		
	Female	32 (8)
	Male	68 (17)
Age at surgery, median (range)		67 (51–83)
Origin of primary tumor, % (n)		
	Pancreas	78 (17)
	Duodenum	12 (3)
	Ampulla of Vater	12 (3)
	Distal common bile duct	8 (2)
Radiological characteristics, % (n)		
	≥ cT3*	28 (7)
	cN1	16 (4)
Neoadjuvant therapy, % (n)		
	Chemotherapy	12 (3)
	Radiotherapy	4 (1)
Adjuvant therapy, % (n)		40 (10)
Preoperative size of tumor (mm), % (n)		
	Not measurable	24 (6)
	≥ 3 cm	36 (9)
	< 3 cm	40 (10)
Laboratory values, median (range)		
	CEA (μg/L)	3.0 (0.2–18.8)
	CA19.9 (kU/L)	132 (3–3426)
	Total bilirubin (μmol/L)	48 (6–376)
	Alkaline phosphatase (U/L)	243 (57–684)
	γ-glutamyl transpeptidase (U/L)	182 (12–2159)

### Averted laparotomies and accuracy of SL

**Fig 2:**
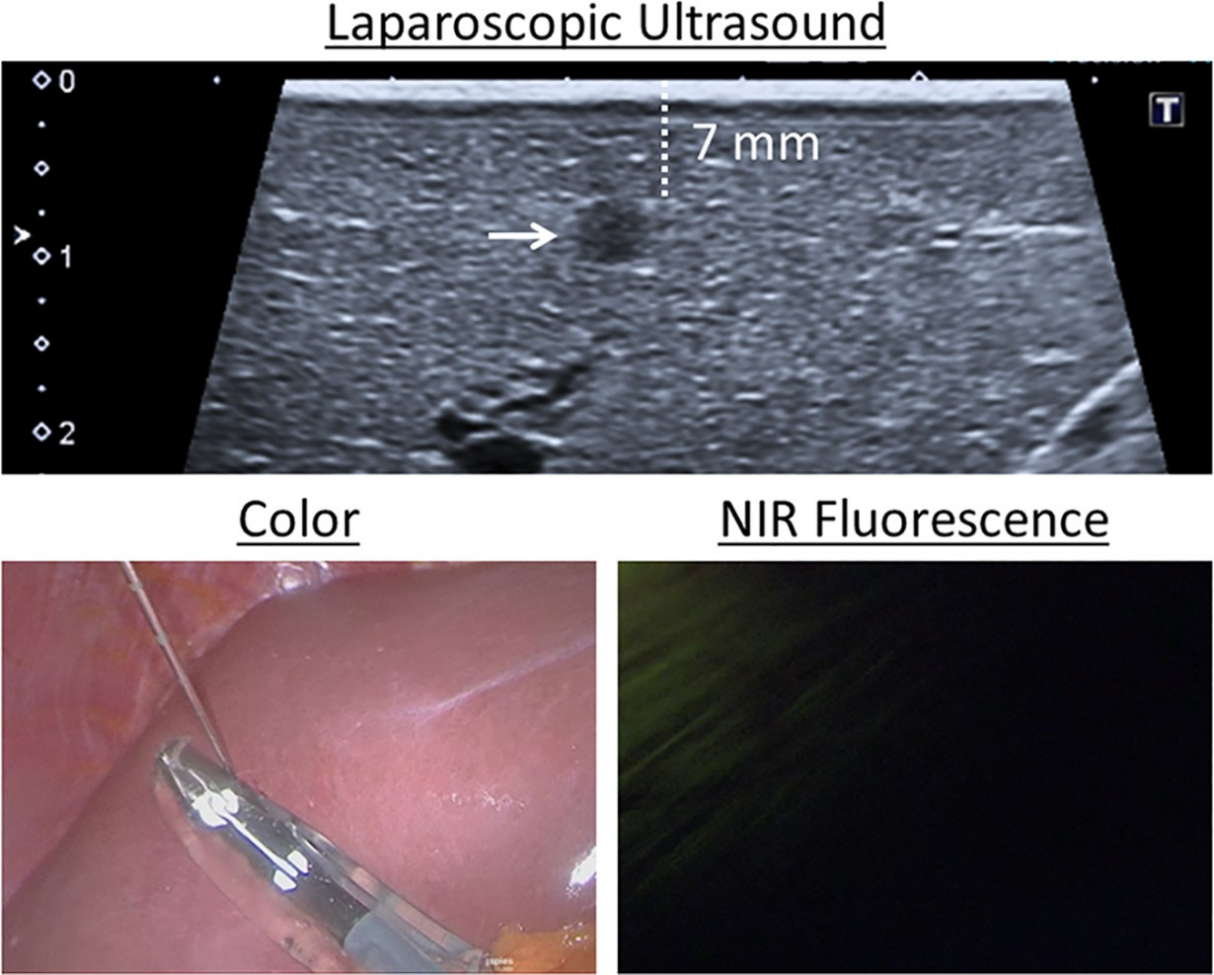
False-positive lesion. A lesion suspected to be a metastasis was detected and biopsied with laparoscopic imaging (arrow). Near-infrared fluorescence imaging did not show any fluorescence signal, even though the lesion was located 7 mm below the liver capsule. The final diagnosis was a bile duct hamartoma.

In 4 patients (16%) the surgeon decided to stop the procedure after completion of the SL due to the detection of apparent metastases. Two patients had liver metastases, which could be identified with inspection, LUS and LFI. One patient had developed peritoneal metastases, which were–as expected—only visible with inspection. In the fourth patient LUS showed a suspect lesion in the liver, while inspection and LFI were negative (Fig 2). Intraoperative frozen section analysis suspected an adenocarcinoma, whereupon the surgical procedure was stopped. The pathological diagnosis was revised postoperatively into a bile duct adenoma after subsequent immunohistochemistry. The patient underwent a laparotomy and resection four days later and remained without metastases up to 4.5 months of follow-up. Thus, in three patients (12%) a futile laparotomy was averted. The positive endpoint (6 averted laparotomies) of this study was therefore not met. Furthermore, all metastases could be identified by inspection, which made LFI and LUS in these cases unnecessary.

Three other suspect lesions were resected in three other patients during SL, but turned out to be benign. All three were assessed as suspect based on inspection only. LFI did not result in false-positive outcomes. Positive and negative predictive values for LFI were 100% (2/2) and 80% (4/5), respectively. Peritoneal metastases were not detected by LFI, but they are never visible since LFI visualizes obstruction of hepatic ICG clearance due to invasive tumor growth.

No new metastases were discovered after laparotomy. In 2 patients (8%) a laparotomy was performed, but the primary tumor appeared to be locally irresectable due to vascular involvement.

### Quality of near-infrared fluorescence imaging

**Fig 3:**
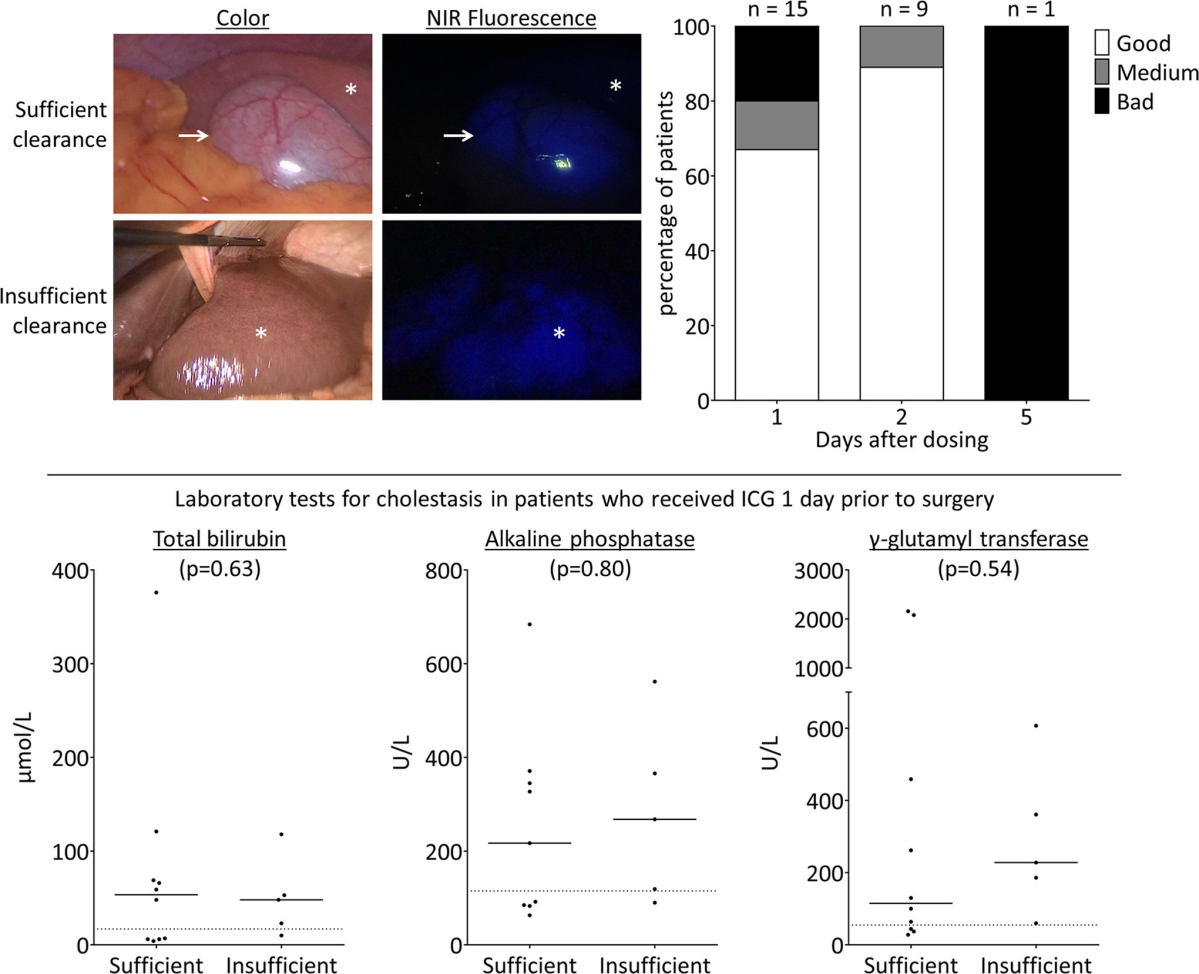
Quality of near-infrared fluorescence imaging. Upper fig: In the majority of patients ICG was cleared sufficiently from healthy liver tissue (star) if administered 2 days before to surgery. The gall bladder (arrow) was used as a positive control. In 33%, the liver still showed significant background fluorescence if ICG was administered 1 day prior to surgery. Lower fig: ICG administered 1 day showed insufficient clearance regardless of cholestatic laboratory values.

Results are shown in Fig 3. Fifteen patients received their dose of ICG one day prior to surgery. In 67% of the patients (n = 10) the quality was good, resulting in sufficient visualization of potential liver lesions. Due to reduced ICG clearance in 20% of the patients (n = 3), the quality was medium, while in 13% (n = 2) the liver was still completely fluorescent.

No significant differences were seen in laboratory tests for cholestasis between patients with sufficient or insufficient quality of LFI. Due to skewed data, Wilcoxon signed-rank tests were performed. ICG was cleared sufficiently by the liver in eight out of nine patients (89%) who were administered ICG two days prior to surgery. The surgical procedure of one patient was postponed after ICG administration with five days due to clinical reasons. During SL no fluorescence signal was detected at all.

### Follow-up

**Fig 4:**
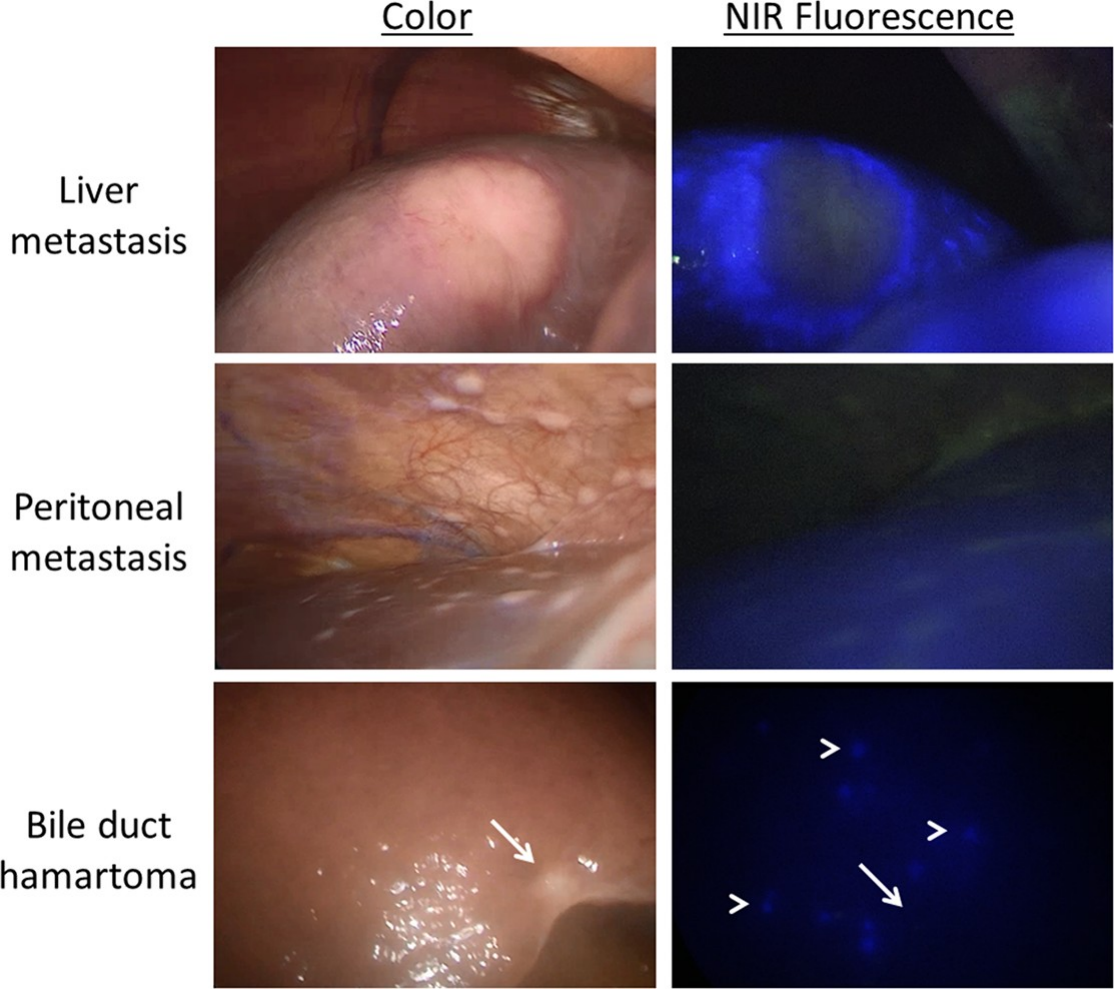
Laparoscopic near-infrared fluorescence imaging. Laparoscopic near-infrared fluorescence imaging could demarcate liver metastases (note the characteristic fluorescent rim), but not peritoneal metastases. Furthermore, discrimination between malignant and benign lesions such as bile duct hamartoma was clear due to the absence of fluorescence in the latter (arrow). In two patients, abnormal fluorescent spots were visible, without a characteristic rim (arrow heads). These lesions were not biopsied, but the patients developed liver metastases within 3 months after surgery.

The median follow-up time of all included patients was 11.8 months (range 1.4–17.5). Liver metastases were diagnosed in 20% (n = 5) of the patients in whom the primary tumor was resected. In two patients, liver metastases were diagnosed within six months after surgery. None were detected during SL or subsequent laparotomy. When including these lesions, accuracies of diagnostic modalities decreased to 44% (4/9), 56% (5/9) and 67% (5/9) for inspection, LUS and LFI, respectively. In hindsight, abnormal fluorescent spots were visible in one patient who developed miliary liver metastases (Fig 4). However, since no suspect fluorescence rim pattern was observed, no biopsies were taken.

## Discussion

Several attempts have been made to increase the yield of SL in pancreatic and periampullary cancer patients, including use of contrast-enhanced ultrasound, LUS, and selection based on preoperative imaging or blood values [8, 15–20]. This is the first study to combine SL with LUS and LFI, but not with the desired result. As mentioned, an unselected population of patients with periampullary cancer was included in this study. This may have resulted in a low *a priori* probability of metastases (12%; 3/25). In hindsight, including only patients with CA 19.9 > 150 U/L or a tumor sized > 3 cm, as suggested in a systematic review covering 24 studies [21], would increase the incidence to 21% (3/14). However, it remains disputable if selection of patients for SL should be performed. During this study period all patients with pancreatic or periampullary cancer who were not included also received SL (without LUS or LFI) before exploratory laparotomy (data not shown). Metastases were detected in 16% (5/31) of these patients. Two of these five patients would not have received SL with the above mentioned selection criteria. Risking that patients with metastases do not undergo SL should be weighed against delaying all procedures with 10 to 15 minutes.

In surgery for colorectal liver metastases, the addition of NIRF imaging to inspection and ultrasound doubled the intraoperative detection rate of additional liver metastases from 13% to 25% [22]. In the current study, we aimed to increase the yield of intraoperative screening for metastases of pancreatic or periampullary cancers by adding fluorescence imaging, but no additional metastases were discovered solely with LFI. One issue could be that, in contrast to patients with colorectal liver metastases, patients with pancreatic or periampullary cancer are more likely to have reduced ICG clearance. Reduced ICG clearance results in nonspecific background fluorescence, which can hamper detection of micrometastases. Our study suggest that within this population dosing ICG two days prior to surgery results in improved quality of fluorescence imaging than one day, regardless of cholestasis laboratory tests.

Apparently, LFI in patients with pancreatic or periampullary cancer has a certain learning curve. During open-space fluorescence imaging, Yokoyama et al. [10] observed abnormal fluorescence spots (larger than 1.5 mm) in 4 patients, but no malignancies could be confirmed by histopathological examination. Three of these patients developed liver metastases within six months. In the current study, only a rim pattern was considered suspect, based on previous experiences with fluorescence imaging of liver metastases [9, 13]. However, abnormal fluorescent spots were seen in two patients (Fig 4) and both developed hepatic metastases shortly after surgery. The yield of LFI may have been higher if also abnormal spots without a rim pattern were analyzed. Yet, LFI was of added value to assess suspect hepatic lesions due to its high negative predictive value; none of the three suspect lesions that turned out to be benign were fluorescent.

LUS was expected to identify intrahepatic metastases and to assess resectability before exploratory laparotomy. However, the technique failed to deliver. Instead, it even resulted in a false-positive biopsy. The added value of LUS is minimized even further when combined with MRI, which is already very sensitive for small, intrahepatic metastases [23]. However, superficial liver metastases remain difficult to distinguish on preoperative imaging. These metastases can be detected with inspection and LFI. The current results suggest that there is no added value of LUS during SL when optimal preoperative imaging has been performed.

In conclusion, this study showed limited added value of LUS during SL in patients with pancreatic or periampullary cancers. Although LFI had a learning curve, it had a high negative predictive value in case of suspect hepatic lesions identified by inspection. If ICG is administered two days prior to surgery it may have more value in a selected patient population.

## Supporting information

S1 File Study protocol in English. (PDF)

S2 File Study data file. (XLSX)

S1 Fig TREND checklist. (PDF)
