# Supplementary material for: Staging laparoscopy with ultrasound and near-infrared fluorescence imaging to detect occult metastases of pancreatic and periampullary cancer
Source: PLoS One. 2018 Nov 1;13(11):e0205960. doi: 10.1371/journal.pone.0205960 (PMC6211678; doi:10.1371/journal.pone.0205960)
Supplement: S1 File — (PDF) [file pone.0205960.s001.pdf]

Amendment X

Protocol P10.001

Staging laparoscopy combined with ultrasonography and near-infrared fluorescence imaging to detect occult pancreatic metastases

Principal investigator: **Dr. J.S.D. Mieog, M.D., Ph.D.**
Leiden University Medical Center
Department of Surgical Oncology
P.O. Box 9600, 2300 RC Leiden
Fax +31 526 6750
E-mail j.s.d.mieog@lumc.nl

Co-investigator: **Dr. A.L. Vahrmeijer, M.D., Ph.D.**
Leiden University Medical Center
Department of Surgical Oncology
P.O. Box 9600, 2300 RC Leiden
Phone +31 71 526 2309
Fax +31 526 6750
E-mail a.l.vahrmeijer@lumc.nl

Co-investigator: **B.A. Bonsing, M.D., Ph.D.**
Leiden University Medical Center
Department of Surgical Oncology
P.O. Box 9600, 2300 RC Leiden
Phone +31 71 526 2309
Fax +31 526 6750
E-mail b.a.bonsing@lumc.nl

Version 3.0 / 08-07-2016

Table of Contents

Table of Contents 2

1 Team members 3

2 Summary 4

3 Background 6

4 Study design 8

5 Objectives 11

5.1 Main objective 11

5.2 Secondary objectives 11

6 Outcomes 11

6.1 Main outcomes 11

- Percentage of averted laparotomies. 11

6.2 Secondary outcomes 11

7 Sample size calculation 12

8 Patient selection criteria 13

8.1 Inclusion criteria 13

8.2 Exclusion criteria 13

9 Reference List 14

1 Team members

Surgery
JSD Mieog
AL Vahrmeijer
BA Bonsing
CJH van de Velde
RJ Swijnenburg
HJM Handgraaf
LSF Boogerd
CES Hoogstins
PJ Bongers

Radiology
AR van Erkel
S Feshtali
MC Burgmans

Anesthesia
H Vuijk
C Martini

Pathology
H Morreau
V Smit
T Bosse

Medical Oncology
SAC Luelmo

2 Summary

**Title**	Staging laparoscopy combined with ultrasonography and near-infrared fluorescence imaging to detect occult pancreatic metastases
**Trial design**	A phase II single center, single-arm trial to assess the added value of staging laparoscopy (SL), laparoscopic ultrasonography (LUS) and near-infrared (NIR) fluorescence imaging in patients with pancreatic cancer undergoing resection with curative intent.
**Rationale**	Even after extensive preoperative assessment, up to 38% of patients undergoing laparotomy with curative intent turn out to have metastases or unresectable disease, preventing curative surgery. Moreover, a substantial number of patients present shortly after surgery with liver metastases that must have been present during surgery, but have not been identified. SL combined with LUS and NIR fluorescence imaging may identify metastases and unresectable disease, sparing patients with incurable disease the morbidity, inconvenience and expense of a futile operation. Staging laparoscopy in pancreatic cancer patients is being advocated in literature, but has not yet been implemented in clinical guidelines.
**Main objective**	The main objective of this study is to determine the added value of SL, LUS and NIR fluorescence imaging in patients with pancreatic cancer undergoing resection with curative intent and to identify predictors for patients that may benefit from SL.
**Endpoints**	- Distant disease-free survival<br>- Overall survival<br>- Percentage of averted laparotomies.<br>- Percentage of averted resections.<br>- Duration of surgical procedures.<br>- Perioperative morbidity and mortality.<br>- Sensitivity of LUS vs. NIR fluorescence imaging.<br>- Positive predictive value of IOUS vs. NIR fluorescence imaging.<br>- Stratification of malignant lesions detected only by NIR fluorescence imaging.
**Patient selection criteria**	Inclusion criteria:<br>- 18 years or older<br>- All patients with pancreatic or periampullary cancer undergoing resection with curative intent.<br>- Absence of any psychological, familial sociological or geographical condition potentially hampering compliance with the study protocol and follow-up schedule; those conditions should be discussed with the patient before

	registration in the trial;<br>- Before patient registration, written consent must be given according to ICH/GCP, national and local regulations.<br>Exclusion criteria:<br>- History of allergy to iodine, shellfish or ICG;<br>- Pregnant or lactating woman;<br>- Any condition that in the opinion of the investigator could potentially jeopardize the health status of the patient.
**Planned trial interventions**	In addition to standard-of-care, patients will receive an intravenous injection of 10 mg ICG 24 prior to surgery. During surgery, patients undergo a SL, whereupon inspection, LUS and NIR fluorescence imaging will be performed. A biopsy will be taken from any suspect lesions. Decision to continue the resection is up to the surgeon. Follow-up will last at least 6 months.
**Statistical design**	Using A'Hern's single stage phase II trial design and alpha=0.05 and power=80%, 25 patients are needed to distinguish between a averted laparotomy rate of 30% (worth exploring in a phase III trial) and 10% or less (unacceptable outcome). Therefore, a total of 25 patients will be included. This requires at least 6 averted laparotomies to reach the positive endpoint.

3 Background

Pancreatic cancer has a very dismal prognosis. Surgery offers the sole chance of long-term survival. However, the presence of metastatic disease in pancreatic cancer patients limits surgical interventions. Even after extensive preoperative assessment, up to 38% of patients undergoing laparotomy with curative intent turn out to have incurable disease, for example due to metastases or an unresectable primary tumor (1). All current preoperative imaging modalities, including CT and MRI, have limited sensitivity in detecting the typically very small peritoneal implants and superficial liver metastases of pancreatic cancer. In a disease with such a poor prognosis, it is important to spare patients with incurable disease the morbidity, inconvenience and expense of a futile operation. Therefore, staging laparoscopy (SL) is being advocated in literature in selected patients (2). However, it has not yet been implemented in clinical guidelines; each individual surgeon has to apply his or her threshold as to whether SL is indicated. One of the aims of this study is to design a clinical protocol for SL that fits in the clinical pathway of patients with pancreatic and periampullary cancer.

The yield of SL depends on the quality of preoperative imaging and the inherent likelihood of metastatic disease (3). In addition, sensitivity of SL can be amplified by adding laparoscopic ultrasonography (LUS) and near-infrared (NIR) fluorescence imaging (4). When no metastatic lesions are found, ultrasonic examination of the whole liver, lymph nodes and superior mesenteric artery can be readily performed. NIR fluorescence imaging using indocyanine green (ICG) is a relatively easy method that can be used to identify metastases in the liver. The hepatic clearance of ICG enable identification of liver tumors, but do not allow detection of metastases located elsewhere. Yokoyama et al. have shown that NIR fluorescence imaging during open surgery identified micrometastases in the liver in 16% (8 of 49) of patients with pancreatic cancer (5). Importantly, abnormal fluorescence of at least 1.5 mm in greatest diameter was highly predictive for manifestation of hepatic metastases within 6 months: 77% (10 of 13) of patients developed hepatic metastases, compared to 3%

(1 of 36) of patients without abnormal fluorescent lesions. Our group showed feasibility of NIR fluorescence imaging of colorectal cancer liver metastases during open surgery (6) and of uveal melanoma liver metastases during laparoscopic procedures (7).

Presently, the only blood-based biomarkers for diagnosis, prognosis and monitoring of treatment in pancreatic patients are measurements of carbohydrate antigen 19-9 (CA19-9) and carcinoembryonic antigen (CEA), but these are not very specific (8). Circulating tumor cells (CTCs) are cells that have detached from the primary tumor, entered the blood circulation and may lead to distant metastases (9). Characterization of CTCs, including analyzing KRAS mutations, may result in further insight into the clinical implications of CTC detection in pancreatic cancer patients.

This study will be the first to combine SL, LUS and NIR fluorescence imaging and aims to determine the added value of these three imaging techniques in patients with pancreatic and periampullary cancer undergoing resection with curative intent. In addition, we aim to identify predictors for patients that may benefit from SL and to design a clinical protocol for SL that fits in the clinical pathway.

4 Study design

All patients planned to undergo explorative or curative surgery for pancreatic or periampullary cancer can be included, provided that they meet the inclusion criteria and none of the exclusion criteria. All patients will receive standard-of-care. This includes a CT-scan < 1 month prior to surgery. Optional imaging, such as (endoscopic) ultrasonography, MRI and FDG-PET, may also be performed if required. In addition, peripheral blood samples (8 mL) will be taken from patients before surgery or chemotherapy. These samples will be analyzed for CTCs, CEA, CA19-9 and bilirubin levels. Patients will receive up to three days before surgery an intravenous injection of 4 ml 2.5 mg/ml ICG (10 mg ICG in total).

The SL procedure starts with visual inspection. The laparoscope is inserted via a subumbilical 10 mm trocar. A second 10 mm port is inserted along the anticipated laparotomy incision to allow LUS under direct vision. Up to two additional 5 mm ports may be necessary to allow exposure of organs and potential palliative surgery. The whole abdomen is inspected, including the parietal and visceral peritoneum, the pelvis, the liver, the porta hepatitis, the gastrohepatic omentum, the duodenum, the transverse mesocolon and celiac region. Secondly, LUS (with or without Doppler) of the primary tumor, liver, portal vein, hepatic artery, lymph nodes and superior mesenteric artery will be performed. NIR fluorescence imaging of the liver surface will be performed lastly. Fluorescence will be divided into rim, partial, full and nonspecific (i.e. artefacts) pattern. Any lesions likely to be metastases are sampled and analyzed, either via a biopsy or via radical resection. In case of multiple lesions with similar appearance on inspection, ultrasound and fluorescence, only one biopsy has to be performed when LUS shows a similar signal in all these lesions. However, when one or more lesions are found solely by NIR fluorescence imaging, multiple small biopsies have to be performed to confirm the diagnosis.

In case no metastases are identified, the surgical procedure will continue, either laparoscopic or via laparotomy. In the validation phase of the laparoscopic fluorescence, the liver will be assessed again with the validated open fluorescence imaging system once the laparotomy has been performed (6). When metastases are identified, the decision to continue the resection of the primary tumor is up to the surgeon. When only NIR fluorescence imaging identifies one or more metastases, the procedure will continue, as the clinical significance of these lesions is yet unknown. Reference for final diagnosis of lesions will be histopathological examination at the pathology department. Furthermore, ascetic fluid will be collected in 3 regions: near the duodenum, the ligament of Treitz and the cavum of Douglas. These samples will be analyzed for tumor cells, similar to the EXPECT study.

Follow-up will occur according to the current local standard protocol, including a visit every three months to the outpatient clinic of surgery in conjunction with the department of oncology. Visit. In short, this includes an outpatient visit to the clinic of surgery every three months. A CT-scan will be performed only in case locoregional or metastatic disease is suspected. Radiologic findings, hematology (e.g. CTCs CA19-9, CEA-mRNA levels), pathology, ascitic fluid etc. will be collected to identify predictors for patients who may benefit from SL.

Risk stratification will be done by stage groups of the tumor and CA19-9 levels.

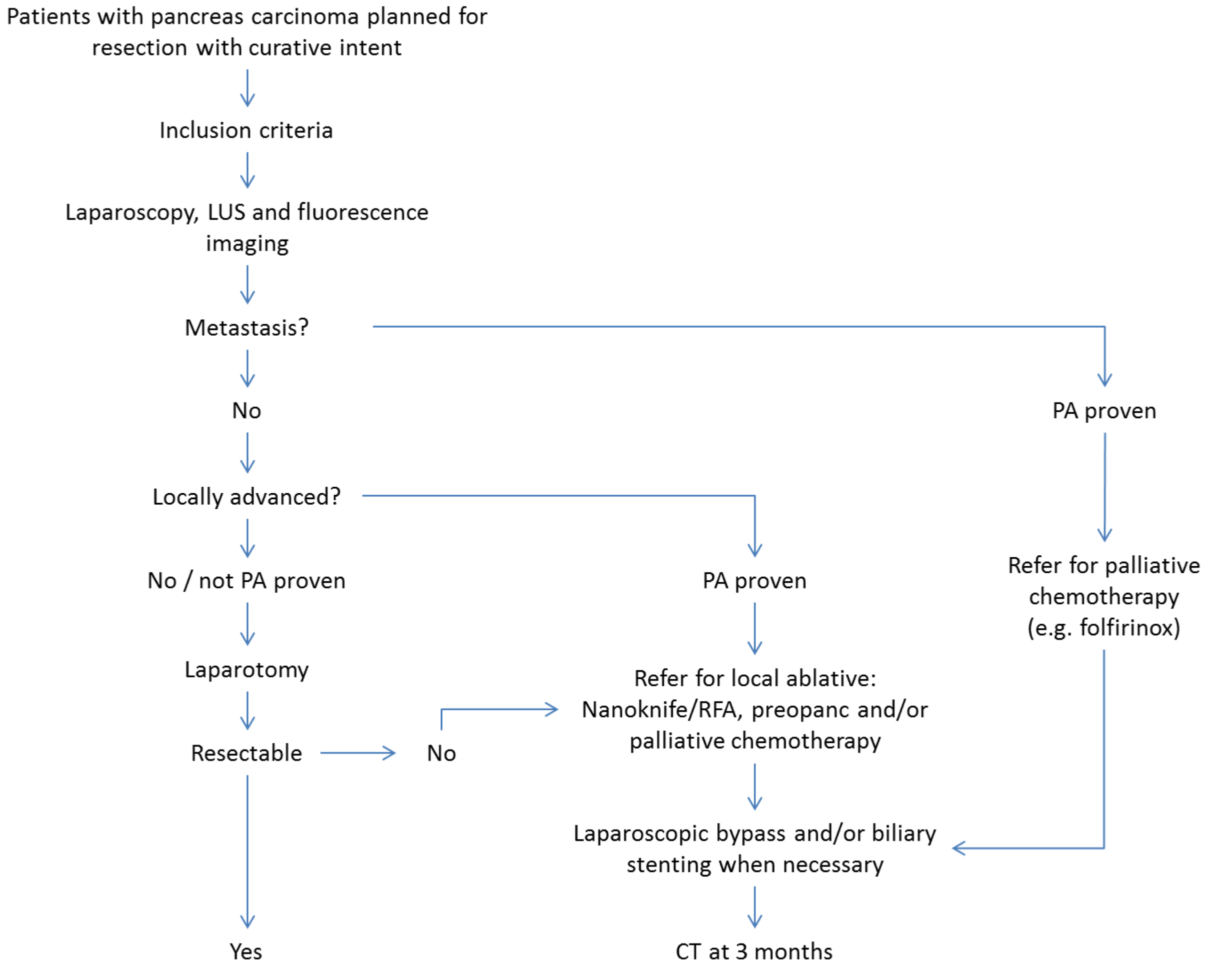

Figure 1. Flow diagram of study. LUS: laparoscopic ultrasonography; PA: pathology; RFA: radiofrequency ablation; CT: computed tomography.

5 Objectives

5.1 Main objective

- To determine if SL, LUS and NIR fluorescence imaging can reduce the laparotomy rate in patients with pancreatic or periampullary cancer planned to undergo resection with curative intent.

5.2 Secondary objectives

- To identify predictors for patients that may benefit from SL.
- To design a clinical protocol for SL that is feasible in the current clinical pathway.

6 Outcomes

6.1 Main outcomes

- Percentage of averted laparotomies.

6.2 Secondary outcomes

- Sensitivity and positive predictive value of laparoscopic inspection vs. LUS vs. NIR fluorescence imaging vs. histopathological examination.
- Positive and negative predictive value of LUS vs. NIR fluorescence imaging on the occurrence of distant metastases.
- Distant disease-free survival (occurrence of distant metastases)
- Overall survival
- Perioperative morbidity and mortality.
- Duration of surgical procedures.

7 Sample size calculation

Using A'Hern's single stage phase II trial design and alpha=0.05 and power=80%, 25 patients are needed to distinguish between a averted laparotomy rate of 30% (worth exploring in a phase III trial) and 10% or less (unacceptable outcome) (10). Therefore, a total of 25 patients will be included. This requires at least 6 averted laparotomies to reach the positive endpoint.

8 Patient selection criteria

8.1 Inclusion criteria

- 18 years or older;
- Patients with pancreatic or periampullary cancer undergoing resection with curative intent;
- Absence of any psychological, familial sociological or geographical condition potentially hampering compliance with the study protocol and follow-up schedule; those conditions should be discussed with the patient before registration in the trial;
- Before patient registration, written consent must be given according to ICH/GCP, national and local regulations.

8.2 Exclusion criteria

- History of allergy to iodine, shellfish or ICG;
- Renal impairment, defined as eGFR > 55;
- Patients with hyperthyroidism;
- Pregnant or lactating woman;
- Any condition that in the opinion of the investigator could potentially jeopardize the health status of the patient.

9 Reference List

(1) Gaujoux S, Allen PJ. Role of staging laparoscopy in peri-pancreatic and hepatobiliary malignancy. World J Gastrointest Surg 2010 Sep 27;2(9):283-90.

(2) Muniraj T, Barve P. Laparoscopic staging and surgical treatment of pancreatic cancer. N Am J Med Sci 2013 Jan;5(1):1-9.

(3) White R, Winston C, Gonen M, D'Angelica M, Jarnagin W, Fong Y, et al. Current utility of staging laparoscopy for pancreatic and peripancreatic neoplasms. J Am Coll Surg 2008 Mar;206(3):445-50.

(4) Handgraaf HJ, Boonstra MC, Van Erkel AR, Bonsing BA, Putter H, Van De Velde CJ, et al. Current and future intraoperative imaging strategies to increase radical resection rates in pancreatic cancer surgery. Biomed Res Int 2014;2014:890230.

(5) Yokoyama N, Otani T, Hashidate H, Maeda C, Katada T, Sudo N, et al. Real-time detection of hepatic micrometastases from pancreatic cancer by intraoperative fluorescence imaging: preliminary results of a prospective study. Cancer 2012 Jun 1;118(11):2813-9.

(6) van der Vorst JR, Schaafsma BE, Hutteman M, Verbeek FP, Liefers GJ, Hartgrink HH, et al. Near-infrared fluorescence-guided resection of colorectal liver metastases. Cancer 2013 Sep 15;119(18):3411-8.

(7) Tummers QR, Verbeek FP, Prevoo HA, Braat AE, Baeten CI, Frangioni JV, et al. First experience on laparoscopic near-infrared fluorescence imaging of hepatic uveal melanoma metastases using indocyanine green. Surg Innov 2015 Feb;22(1):20-5.

(8) Mehta J, Prabhu R, Eshpuniyani P, Kantharia C, Supe A. Evaluating the efficacy of tumor markers CA 19-9 and CEA to predict operability and survival in pancreatic malignancies. Trop Gastroenterol 2010 Jul;31(3):190-4.

(9) Tjensvoll K, Nordgard O, Smaaland R. Circulating tumor cells in pancreatic cancer patients: methods of detection and clinical implications. Int J Cancer 2014 Jan 1;134(1):1-8.

(10) A'Hern RP. Sample size tables for exact single-stage phase II designs. Stat Med 2001 Mar 30;20(6):859-66.
